# Rapid detection of newly isolated Tembusu-related Flavivirus by reverse-transcription loop-mediated isothermal amplification assay

**DOI:** 10.1186/1743-422X-8-553

**Published:** 2011-12-21

**Authors:** Youling Wang, Xiaoyuan Yuan, Yufeng Li, Kexiang Yu, Jinxing Yang, Huaiying Xu, Yuxia Zhang, Kangzhen Yu, Ming Liao, Zhuoming Qin

**Affiliations:** 1 College of Veterinary Medicine, South China Agricultural University, Guangzhou 510642, PR China; 2 Institute of Poultry Science, Shandong Academy of Agricultural Science, Jinan 250023, PR China; 3 Ministry of Agriculture of the People's Republic of China, Beijing 100026, PR China

**Keywords:** Tembusu-related Flavivirus, newly-isolated virus, RT-PCR, RT-LAMP

## Abstract

**Background:**

From April 2010 to January 2011, a severe new viral disease had devastated most duck-farming regions in China. This disease affected not only laying ducks but also meat ducks, causing huge economic losses for the poultry industry. The objective of this study is to develop a one-step reverse transcription loop-mediated isothermal amplification (RT-LAMP) assay for the detection of the new virus related to Tembusu-related Flavivirus.

**Results:**

The RT-LAMP assay is very simple and rapid, and the amplification can be completed within 50 min under isothermal conditions at 63°C by a set of 6 primers targeting the E gene based on the sequences analysis of the newly isolated viruses and other closely related Flavivirus.The monitoring of gene amplification can also be visualized by using SYBR green I fluorescent dye. In addition, the RT-LAMP assay for newly isolated Tembusu-related Flavivirus showed higher sensitivity with an RNA detection-limit of 2 copies/μL compared with 190 copies/μL of the conventional RT-PCR method. The specificity was identified without cross reaction to other common avian pathogens. By screening a panel of clinical samples this method was more feasible in clinical settings and there was higher positive coincidence rate than conventional RT-PCR and virus isolation.

**Conclusion:**

The RT-LAMP assay for newly isolated Tembusu-related Flavivirus is a valuable tool for the rapid and real-time detection not only in well-equipped laboratories but also in general conditions.

## Introduction

In April 2010, a severe viral disease spread out in most duck-farming regions in China including Zhejiang, Jiangsu, Hebei and Shandong provinces. The disease affected both meat ducks and laying ducks. The affected layer ducks showed clinical symptom of heavy egg-laying decrease ranging from 20% to 60%, even 90% [1].During the course of disease, some ducks developed neurological signs including unsteady standing, falling and quivering to death. In some young-duck farms, the disease developed as early as in 10-day-old ducklings, with a peak in 20-40 days old ducklings. The main symptoms were unable to stand steadily and falling. The death rate was normally within 10-30%, and could be as high as 80%, causing huge economic losses in duck-farming. We isolated some apparently new flavivirus from duck incubated in 10-day-old SPF chicken embryos and duck embryos, and found that the isolates belonged to the genus flavivirus based on sequence analysis. The virus had lower nucleotide homology with other genus of flavivirus.

In consideration of biological characters and clinical symptoms, we suggested Duck Encephalitis virus (DEV) was named for this newly virus. DEV infection can cause duck encephalitis with severe central nervous system disorders and egg laying decrease. Diagnosis of DEV infection is mainly based on viral culture and molecular approaches [2]. Virus isolation is a definitive diagnosis for DEV infection, however, this assay is usually unfeasible owing to less sensitive and time-consuming. RT-PCR assays for detection of specific genomic sequence of DEV have shown high sensitivity and specificity. However, these assays are time-consuming and require expensive and sophisticated equipments [3]. DEV often occur in rural areas where routine RT-PCR diagnostic facilities are limited. Therefore, it is necessary for us to develop a rapid, simple, sensitive, and specific diagnostic method for DEV infection and surveillance.

LAMP is a novel PCR method and its most important advantage is amplification of nucleic acids under isothermal conditions at a temperature range between 60 and 65°C within 1 h [4-6]. Another advantage of the method is that the amplification can lead to the accumulation of large amounts of products of various lengths, making detection of amplified nucleic acids much easier[7]. Recently, LAMP technology has been successfully applied for rapid detection of various pathogens [8-12]. A RT-LAMP assay for detecting JEV was firstly reported by Toriniwa and Komiya and the sensitivity was similar to conventional RT-PCR [13]. Later, Parida used the application of RT-LAMP assay to detect JEV in the cerebrospinal fluid samples from patients with clinical diagnosis of acute encephalitis [14].

So far, there has been no report on using the RT-LAMP to rapidly diagnose and identify DEV. In this study, we established the RT-LAMP method to rapidly diagnose and identify DEV. The assay can be a new standard for the virus identification.

## Methods

### Design of DEV specific RT- LAMP primers

Based on the sequence of newly isolated Tembusu-related Flavivirus BYD-1 published in GenBank (Genbank accession no.JF312912.1) and other unpublished sequences that we collected, a highly conserved region of the E gene was chosen to design the RT-LAMP primers. A set of 6 primers was designed comprising two outer (F3, B3), two inner (FIP, BIP) and two loop primers (FLP, BLP), which could recognize eight distinct regions on the target sequence by software program (http://primerexplorer). FIP and BIP were high performance liquid chromatography-purified primers. FLP and BLP primers were composed of the sequences that were complementary to the sequence between F1 and F2, and between B1 and B2 regions, respectively. All primers were composed by Shanghai BGI Company. The details of the primers with regard to their positions in the genomic sequences were shown in Table 1.

**Table 1:** Details of RT-LAMP primers

Primer name and component	Position	Sequence
F3	(1039-1058)	AATGACATGACACCAGTTGGGCA ACCATCCTTTGTGCTC
B3	(1259-1241)	ATGGAGGTTCCACTTCCACCT
FIP(F1c+TTTT+F2)	(1135-1120+TTTT+1069-1087)	TTTACAGTCAACCCATACGTGT GGTAGGAAGTGGAAAAGGACTTTT TAAAAGCTTTTCCAATTGT
BIP(B1c+TTTT+B2)	(1155-1174+TTTT+1225-1207)	GGCACCCGTGGAGGAGGTCG AGATCAGGTACCAGTGGCATAG
FLP	(1106-1088)	
BLP	(1175-1196)	

### Clinical samples

Three new DEV were isolated from layer ducks with typical eggs-laying decrease symptoms and young duck with neurological signs from 2010 to 2011 by using 10-day-old SPF chicken(duck) embryonated eggs, which were confirmed as DEV by sequencing. All of the isolates were stored at -80°C until further investigation. Their alignment analysis of nucleotide homology showed that the new isolates belonged to the genus flavivirus, Ntaya virus group. In addition, the control viruses included duck plague virus(DPV) isolate NJ, duck hepatitis virus (DHV), low-pathogenicity avian influenza virus (LP-AIV) H9N2 and swine encephalitis vaccine virus SA-14, all were obtained from the Poultry Institute of Shandong.

### RNA extraction

The genomic viral RNA was extracted from the cultures obtained from embryo by using the MiniBEST viral RNA Extraction kit (TaKaRa, Japan) according to the manufacturer's protocol. The RNA was eluted in a final volume of 50 μL of elution buffer and stored at -80°C until further use.

### RT-PCR

In order to compare the sensitivity and specificity of the RT-LAMP assay, one-step RT-PCR was performed with DEV-specific primers designed from the E gene (Genbank accession no.JF312912.1). E-F: 5^, ^CCACGGAATTAGCGGTTGT3^, ^(position no.149 to 167), and E-R: 5^,^TAAGTTGCCTTGGGATTATGAG 3^, ^(position no. 261 to 279), targeting 112 bp. The amplification was done in a reaction volume of 50 μL by using the TaKaRa One-Step RT-PCR kit with 50 pmol of forward and reverse primers and 2 μL of RNA according to the manufacturer's protocol. The thermal profile of RT-PCR was 50°C for 40 min and 94°C for 2 min, followed by 32 cycles of 94°C for 30 s, 52°C for 30 s, 72°C for 30 s, and a final extension cycle of 72°C for 10 min.

### RT-LAMP

The RT-LAMP reaction was done in a reaction volume of 25 μL containing 40 pmol each of the primers FIP and BIP, 5 pmol each of the outer primers F3 and B3, 20 pmol each of the loop primers FLP and BLP, 1.0 mM deoxynucleoside triphosphate (Promega), 0.8 M betaine (Ferments), 8 U of Bsm DNA polymerase (Ferments), 10 U of the avian myeloblastosis virus reverse transcriptase (TaKaRa), and 2 μL of the target RNA. The reaction mixture was incubated at 63°C for 50 min in a heating block and followed by heating at 85°C for 2 min to terminate the reaction. Negative and positive controls were done in each run, and all precautions were adopted to prevent cross-contamination.

### Sensitivity and specificity of RT-LAMP and RT-PCR

The specificity of RT-LAMP reaction was done for the three isolated strains. DPV isolate NJ, DHV, H9N2 and SA-14 were used as contrastive specimens. The sensitivity of the RT-LAMP assay for the detection of DEV RNA was determined by testing serial 10-fold dilutions of RNA and compared with that of conventional RT-PCR. In order to evaluate the detection limit of the assays, the amount of DEV RNA was determined by spectrophotometer and converted to molecular copies by using the following computational formula [15].

$$\text{Y(molecules/ul)}=\frac{\text{X(g/ul)RNA}}{\text{transcriptlength(bp)}\times 340}\times 6.02\times 10^{23}$$

Y is molecular copies, X is optical density ratio.

### RT-LAMP visualization

The monitoring of RT-LAMP amplification was visually observed under UV light (302 nm) following the addition of 1 μL of SYBR green I (1:1000) dye to the tube.

### Agarose gel analysis

After incubation at 63°C for 50 min, 10 μL RT-LAMP product was electrophoresed on a 2% agarose gel in TAE buffer, followed by staining with ethidium bromide and observed in image-forming system.

### RT-LAMP assay with virus-attacked samples

15-day-old ducks were randomly divided into 2 groups: the first group of 10 as drug group with an attacking agent volume of 10^5^TCID_50 _ml^-1 ^by calculating with the Reed-Muench cumulative method. The second group was the control group, attacked by the same volume of saline injection. Mortality was recorded daily for 10 days. After 10 days, dead ducks (7), ill ducks (3) and the control ducks were killed and some organs (liver, kidney, spleen, brain, follicle membrane) were processed as samples for RT-LAMP.

### RT-LAMP assay with clinical samples

The applicability of the RT-LAMP assay for clinical diagnosis of DEV was validated with possible samples collected from China between 2010 and 2011, and the results were compared with those of conventional RT-PCR and virus isolation. A total of 88 clinical samples after clinic examination and serological testing were used in this study for comparison.

## Results

**Figure 1:**
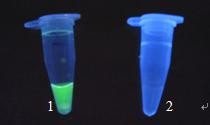
**The inspection of the RT-LAMP reaction stained with SYBR Green I under UV light**. Tubes 1: DEV SD isolate showed green color; Tube 2: JEV SA14 did not show green color.

Following RT-LAMP amplification, white turbidity was visually observed in the bottom of the tube. The inspection for amplification was also performed through the observation of colour change following the addition of 1 μL of SYBR green I (1:1000) dye to the tube. For positive amplification, the original orange colour of the dye changed to green that can be judged under UV light (302 nm) (Figure 1).

**Figure 2:**
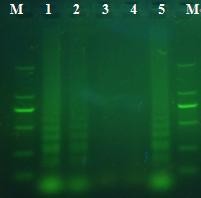
**Amplified products of the RT-LAMP assay were observed on a 2% agarose gel electrophoresis under UV**. Three DEV positive amplifications showed a ladder-like pattern (lane 1,2,5). M: marker DL2000; 1:BZ isolate;2:LC isolate; 3: DPV NJ; 4: JEV SA14;5: SD isolate.

**Figure 3:**
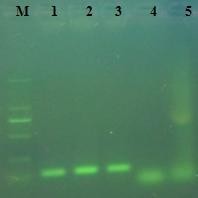
**Amplified products of the RT-PCR assay are observed on a 2% agarose gel electrophoresis under UV**. Three DEV positive amplifications showed 112bp band (lane 1,2,3). M: marker DL2000;1:BZ isolate; 2:LC isolate;3:SD isolate; 4: DPV NJ; 5: JEV SA14.

As observed on agarose gel electrophoresis, the RT-LAMP assay could amplify the target sequence of the E gene of DEV at 63°C in 50 min. The amplification was observed as a ladder-like pattern on the gel due to the formation of a mixture of stem-loop DNAs with various stem lengths (Figure 2). The RT-PCR could amplify the target sequence on agarose gel electrophoresis (Figure 3).

### Sensitivity and specificity of RT-LAMP and RT-PCR

The DEV-specific RT-LAMP primers demonstrated a high degree of specificity for DEV only and failed to detect all the control viruses. Further confirmation of the structures of the amplified products was also done by sequencing. The obtained sequences perfectly matched with the expected nucleotide sequences.

The sensitivity of the RT-LAMP assay for the detection of DEV RNA was determined by testing serial 10-fold dilutions of viral RNA and compared with that of conventional RT-PCR.

**Figure 4:**
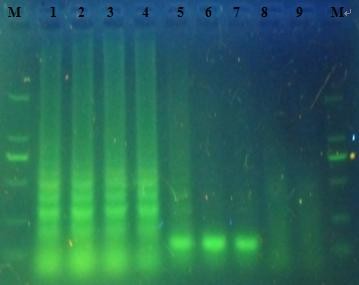
**Amplified products of the RT-LAMP and RT-PCR assay were observed on a 2% agarose gel electrophoresis under UV in the sensitivity of the RT-LAMP assay**. M: marker DL2000;1-5: Amplified products of serial 10-fold dilutions of virus in RT-LAMP: 1.9 × 10^5^, 1.9 × 10^4^, 1.9 × 10^3^, 1.9 × 10^2^, 1.9 × 10^1 ^copies of RNA, respectively; 6-9: Amplified products of serial 10-fold dilutions of virus in RT-PCR 1.9 × 10^5^, 1.9 × 10^4^, 1.9 × 10^3^, 1.9 × 10^2^copies of RNA, respectively.

The RT-LAMP assay was able to amplify the 10^-5 ^fold RNA, and the comparative sensitivity of RT-PCR revealed that it was 10^-2 ^fold with the same original RNA. RNA initial concentration was converted to 1.9×10^5 ^copies/μL according to above calculation formula. So the detection limit of the RT-LAMP assay was 2 copies/μL whereas the detection limit of the RT-PCR assay was 190 copies/μL under UV light with dying (Figure 4). Therefore, RT-LAMP assay was more sensitive than RT-PCR. Furthermore, RT-LAMP can complete within 1 h, much faster than that of RT-PCR (3 h).

### RT-LAMP assay with virus-attacked samples

The organs from the dead ducks (7), ill ducks (3) showed special LAMP light through RT-LAMP assay. Samples such as liver, kidney, spleen, brain and follicle membrane were all confirmed by DEV attacking. There were no reactions observed for the control ducks.

### Evaluation of DEV RT-LAMP assay with clinical samples

A total of 88 clinical samples (including liver, kidney, spleen, brain and follicle membrane) after clinic examination were used in this study. The comparative evaluation of RT-LAMP and one-step RT-PCR revealed that RT-LAMP had higher positive coincidence rate. As summarized in Table 2, percentage of positive samples detected by conventional RT-PCR, virus isolation and RT-LAMP were 81/88, 74/88 and 85/88, respectively.

**Table 2:** Detection positive rates of clinical specimens by RT-PCR, RT-LAMP and virus isolation

Source(N0) \ Assay	RT-PCR	RT-LAMP	Virus isolation
Liver(20)	90.0%	95.0%	80.0%

Kidney(18)	75.0%	94.4%	77.8%

Spleen(18)	94.4%	100%	94.4%

Brain(16)	93.8%	93.8%	81.3%

Follicle membrane(16)	100%	100%	87.5%
Positive rate(%)	92.0%	96.6%	84.1%

The RT-LAMP assay was evaluated to detect four additional positive cases, whereas one-step RT-PCR failed to detect. The sequencing of these 4 additional cases matched with the expected nucleotide sequences, thereby indicating the superior sensitivity of RT-LAMP assay.

## Discussions

The LAMP method does not rely on expensive and sophisticated facilities such as thermal cyclers. The amplified products of the reaction are shown as a ladder-like pattern by agarose gel electrophoresis. The products could be visually observed under UV light or normal light by adding fluorescent dye. A by-product of the LAMP reaction, magnesium pyrophosphate, can be directly inspected by naked eye. The ordinary PCR methods require either high-precision instruments for the amplification or elaborate methods for the detection of the amplified products [16]. In addition, these methods are often cumbersome to adapt for routine clinical use, especially in peripheral health care settings and private clinics [17].

Here, we described the establishment of a one-step, single tube RT-LAMP assay for rapid detection of the envelope gene of DEV genome and compared its sensitivity and specificity with conventional RT-PCR. The RT-LAMP assay for DEV showed excel specificity in positive viruses and contrastive strains. As already shown in the results, the estimated detection limit (2 copies/μL) of the DEV RT-LAMP assay was more sensitive than conventional RT-PCR with the estimated detection limit of 190 copies/μL.

## Conclusions

In conclusion, this study presented a simple, sensitive and specific RT-LAMP assay for detection of specific nucleic acid sequence of DEV E gene. Considering these advantages, the RT-LAMP assay can be applied as a practical molecular diagnostic tool for DEV infection and surveillance in laboratory or general conditions.

## Competing interests

The authors declare that they have no competing interests.

## Authors' contributions

YW,XY,YL,KY,JY,HX,YZ carried out the experiments and wrote the manuscript. XY performed the statistical analysis. KY,ML,ZQ participated in experimental design and coordination. All authors read and approved the final manuscript.

## References

[B1] Jingliang Su Shuang Li Xudong Hu Xiuling Yu Wang Yongyue Peipei Liu Xishan Lu Zhang Guozhong Xueying Hu Di Liu Xiaoxia Li Wenliang Su Hao Lu Shing Mok Ngai Wang Peiyi Wang Ming Tian Kegong George F Gao: Duck Egg-Drop Syndrome Caused by BYD Virus, a New Tembusu-Related Flavivirus PLoS ONE in press 10.1371/journal.pone.0018106 PMC3063797 21455312

[B2] Yufeng Li Xiuli Ma Kexiang Yu Youling Wang Gao Wei Bing Huang Huangying Xu Jing Wu Shengyu Wang Lili Wang Zhuoming Qin A brief report of flaviviruses newly isolated from duck Acta Veterinaria et Zootechnica Sinica 2011 6 885 891

[B3] Burke DS Nisalak A Ussery MA Antibody capture immunoassay detection of Japanese encephalitis virusimmunoglobulin M and Gantibodies in cerebrospinal fluid J Clin Microbio 2002 16 1034 1042 10.1128/jcm.16.6.1034-1042.1982 PMC272535 7161371

[B4] Chen HT Zhang J Sun DH Ma LN Liu XT Cai XP Liu YS Development of reverse transcription loop-mediated isothermal amplification for rapid detection of H9 avian influenza virus J Virol Methods 2008 151 200 203 10.1016/j.jviromet.2008.05.009 18572258

[B5] Kiatpathomchai W Jareonram W Jitrapakdee S Flegel TW Rapid and sensitive detection of taura syndrome virus by reverse transcription loop-mediated isothermal amplification J Virol Methods 2007 146 125 128 10.1016/j.jviromet.2007.06.007 17643501

[B6] Le Roux CA Kubo T Grobbelaar AA Jansen van Vuren P Weyer J Nel LH Swanepoel R Morita K Paweska JT Development and evaluation of a real-time reverse transcription-loop-mediated isothermal amplification assay for rapid detection of rift valley fever virus in clinical specimens J Clin Microbiol 2009 47 645 651 10.1128/JCM.01412-08 19109471 PMC2650915

[B7] Li Q Zhou QF Xue CY Ma JY Zhu DZ Cao YC Rapid detection of porcine reproductive and respiratory syndrome virus by reverse transcription loop-mediated isothermal amplification assay J Virol Methods 2009 155 55 60 10.1016/j.jviromet.2008.09.012 18926852

[B8] Peyrefitte CN Boubis L Coudrier D Bouloy M Grandadam M Tolou HJ Plumet S Real-time reverse-transcription loop-mediated isothermal amplification for rapid detection of rift valley fever virus J Clin Microbiol 2008 46 3653 3659 10.1128/JCM.01188-08 18799705 PMC2576582

[B9] Dukes JP King DP Alexandersen S Novel reverse transcription loop-mediated isothermal amplification for rapid detection of foot-and-mouth disease virus Arch Virol 2006 151 1093 1106 10.1007/s00705-005-0708-5 16453084

[B10] Chen C Cui S Zhang C Li J Wang J Development and validation of reverse transcription loop-mediated isothermal amplification for detection of PRRSV Virus Genes 2010 40 76 83 10.1007/s11262-009-0419-1 19911264

[B11] Xu JT Zhang ZM Yin YB Cui SJ Xu SZ Guo YY Li JD Wang JL Liu XC Han LM Development of reverse-transcription loop-mediated isothermal amplification for the detection of infectious bursal disease virus J Virol Methods 2009 162 267 271 10.1016/j.jviromet.2009.07.010 19643144

[B12] Xue CY Zhang Y Zhou QF Xu C Li XM Cao YC Rapid detection of Infectious bursal disease virus by reverse transcription loop-mediated isothermal amplification assay J Vet Diagn Invest 2009 21 841 843 10.1177/104063870902100612 19901286

[B13] Toriniwa H Komiya T Rapid detection and quantification of Japanese encephalitis virus by real-time reverse transcription loop-mediated isothermal amplification Microbiol Immunol 2006 50 379 387 16714845 10.1111/j.1348-0421.2006.tb03804.x

[B14] Parida MM Santhosh SR Dash PK Tripathi NK Saxena P Ambuj S Sahni AK Lakshmana Rao PV Morita K Development and evaluation of reverse transcription-loop-mediated isothermal amplification assay for rapid and real-time detection of Japanese encephalitis virus J Clin Microbiol 2006 44 4172 4178 10.1128/JCM.01487-06 17005741 PMC1698363

[B15] Russell Sambrook Molecular cloning 2002 3 Cold Spring Harbor Lab Press 1695 1697

[B16] Nagamine K Watanabe K Ohtsuka K Hase T Notomi T Loop-mediated isothermal amplification reaction using a nondenatured template Clinical Chemistry 2001 47 1742 1743 11514425

[B17] Kaneko H Kawana T Fukushima E Suzutani T Tolerance of loop-mediated isothermal amplification to a culture medium and biological substances Journal of Biochem and Biophys Methods 2007 70 499 501 10.1016/j.jbbm.2006.08.008 17011631

